# Probiotics in Poultry: A Comprehensive Review of Mechanisms, Applications, and Future Directions

**DOI:** 10.3390/vetsci13080831

**Published:** 2026-08-19

**Authors:** Zhe Jia, Yanfei He, Haijun Xu, Cai Zhang, Shunan Cuan

**Affiliations:** 1 College of Life and Health, West Anhui University, Lu’an 237012, China; 2 Henan International Joint Laboratory of Animal Welfare and Health Breeding, Henan University of Science and Technology, Luoyang 471023, China

**Keywords:** probiotics, poultry, gut microbiota, antibiotic alternatives, intestinal health

## Abstract

Global poultry consumption continues to rise, while worldwide bans on in-feed antibiotic growth promoters raise an urgent requirement for eco-friendly alternatives to guarantee production efficiency and food safety. Probiotics, live beneficial microorganisms that improve host intestinal health, are reviewed. We elaborate four core functional pathways of probiotics: competitive exclusion of pathogens, enhancement of intestinal barrier integrity, immune modulation and regulation of microbial metabolites such as short-chain fatty acids. Their mitigating effects against heat stress, suboptimal rearing environments, mycotoxin contamination, heavy metal exposure and immune stress are analyzed. We further evaluate the capacity of single and compound probiotics to control major poultry diseases. Early-life intervention strategies and innovative preparations (multistrain probiotics, synbiotics, postbiotics) are systematically summarized. Critical bottlenecks restricting industrial translation are highlighted, including empirical strain combination, non-standardized administration protocols, divergent evaluation indicators and single-factor laboratory challenge models inconsistent with actual farm conditions. Finally, we propose future research directions covering multi-omics-assisted strain screening, optimized delivery technology, unified industrial quality control standards and field verification under compound stress. This review offers integrated references for mechanistic research, strain development and precise industrial application of probiotics in sustainable antibiotic-free poultry breeding.

## Simple Summary

Many countries have banned antibiotic growth promoters in poultry feed, creating an urgent demand for safe and sustainable alternatives. Probiotics are one of the most promising substitutes to maintain poultry gut health and production performance. This review summarizes how probiotics protect chickens by suppressing harmful bacteria, repairing intestinal barriers and regulating immunity, and outlines their ability to relieve heat stress, mycotoxin damage and other common farming challenges. We also discuss the practical value of probiotics for preventing necrotic enteritis, salmonellosis and other bacterial diseases, as well as advanced forms including multistrain mixtures, synbiotics and postbiotics. At present, inconsistent on-farm results limit large-scale probiotic application, which is mainly caused by blind strain matching, lack of unified usage standards and oversimplified laboratory research models. We put forward targeted research ideas based on multi-omics screening, unified evaluation systems and field trials under complex stress conditions. This paper provides clear theoretical guidance for developing and using probiotic products in antibiotic-free poultry production.

## 1. Introduction

Global demand for poultry products has grown substantially over recent decades, with chicken consumption alone increasing by approximately 70% in 30 years, placing pressure on the industry to lift production efficiency while maintaining food safety and animal welfare [1,2]. Antibiotic growth promoters (AGPs) were once widely added to poultry feed to improve feed conversion and reduce mortality, but their long-term use has triggered serious public health risks such as antimicrobial resistance, drug residues, and gut dysbiosis [3]. Many countries have now restricted or banned in-feed AGPs, creating an urgent need for effective, safe, and eco-friendly alternatives for sustainable poultry production [1,2].

Among the various alternatives explored, probiotics have emerged as one of the most promising candidates for replacing AGPs in poultry production [3,4]. Probiotics are defined as live microorganisms that, when administered in adequate amounts, confer a health benefit on the host [3,5]. From probiotics to postbiotics: Concepts and applications. They exert their beneficial effects through multiple mechanisms, including competitive exclusion of pathogenic bacteria, production of antimicrobial substances, enhancement of intestinal barrier integrity, modulation of host immunity, and improvement of digestive enzyme activity [3]. By restoring or maintaining gut homeostasis, probiotics can improve feed conversion efficiency, body weight gain, carcass yield, and egg production while reducing the incidence of intestinal infections [6]. The outcomes of probiotic supplementation have not always been consistent across studies, with variability attributed to differences in bird age, breed, housing conditions, health status, probiotic strain composition, dosage, and administration strategy [7].

Accordingly, this review provides a synthesis of poultry probiotic research from a gut microbiota–host interaction perspective. Beyond cataloging reported effects, we systematically compare efficacy across representative strains, application scenarios, and administration strategies. We further evaluate the translational potential of novel formulations and propose actionable future directions to bridge laboratory findings and industrial practice.

## 2. Literature Search Strategy

This review is based on peer-reviewed publications retrieved from three major databases: PubMed, Web of Science, and CNKI. The search covered studies published from 2010 to 2025, using combinations of the following keywords: (“probiotics” OR “synbiotics” OR “postbiotics”) AND (“poultry” OR “broiler” OR “layer” OR “chicken”). Inclusion criteria were (1) original research articles conducted in poultry models; (2) studies evaluating the effects of probiotic supplementation on production performance, health status, or disease resistance; and (3) studies with clear strain identification and dosage information.

## 3. Mechanisms of Probiotic Action in Poultry

The gastrointestinal tract of poultry harbors a dense and diverse microbial community that exerts profound effects on host physiology, nutrient metabolism, immune development, and resistance to pathogen colonization [8]. Rather than relying on a single mode of action, probiotic strains exert their influence through direct antagonism of pathogens, modulation of the host immune system, reinforcement of intestinal barrier integrity, and production of bioactive metabolites (Table 1). Understanding these mechanisms is essential for rational strain selection and for predicting in vivo efficacy across poultry and physiological states.

### 3.1. Competitive Exclusion and Pathogen Antagonism

One of the most well-characterized mechanisms of probiotic action is competitive exclusion, whereby probiotic microorganisms compete with pathogenic bacteria for adhesion sites and nutrients within the gut ecosystem [4,9,10]. By occupying ecological niches and consuming available substrates, probiotics can prevent the colonization and proliferation of enteric pathogens such as *Salmonella*, *Escherichia coli*, *Clostridium perfringens*, and *Campylobacter* species [10,18]. This mechanism is particularly relevant in young animals with immature gut microbiota, where the establishment of beneficial microbial communities can confer early protection against infectious challenges [21,25].

In addition to passive competition, many probiotic strains actively produce antimicrobial substances that directly inhibit or kill pathogenic microorganisms. These include organic acids (e.g., lactic acid, acetic acid, propionic acid), which lower the luminal pH and create an unfavorable environment for acid-sensitive pathogens [9,12]. Class II bacteriocins produced by lactic acid bacteria, including sakacin A, pediocin PA-1, enterocins, plantaricins, and garvicin ML, have been shown to inhibit potentially problematic bacteria such as *Staphylococcus*, *Enterococcaceae*, and *Clostridium* in the mouse gut, while simultaneously increasing the proportion of lactic acid bacteria [13]. Lactic acid bacteria isolated from the poultry gastrointestinal tract have demonstrated potent antagonistic activity against a broad spectrum of pathogens, including *E. coli* and its serotype O157:H7, *Enterococcus faecalis*, *Salmonella typhimurium*, *Salmonella enteritidis*, and *Listeria monocytogenes*, with zones of inhibition ranging from 12.5 to 20 mm [11]. Selected strains of *Limosilactobacillus reuteri*, *P. acidilactici*, *P. pentosaceus*, and *Enterococcus faecium* also competitively excluded these pathogens while adhering to ileum epithelial cells [11].

Beyond bacteriocins, *Bacillus* spp. produce a diverse arsenal of antimicrobial metabolites. *B. subtilis* KATMIRA1933 and *Bacillus amyloliquefaciens* B-1895, which produce the bacteriocins subtilosin A and subtilin, respectively. They inhibit biofilm formation by multiple *Salmonella enterica* serovars without affecting planktonic cell numbers, suggesting that quorum sensing disruption rather than direct killing mediates this effect [14]. Similarly, *B. subtilis* natural isolate PS-216 prevents adhesion and biofilm development of *Campylobacter jejuni* on abiotic surfaces and can destroy preestablished biofilms, an activity attributed to the polyketide compound bacillaene [15].

### 3.2. Enhancement of Intestinal Barrier Integrity

The intestinal epithelium serves as a critical physical and immunological barrier that separates the luminal contents from the host internal environment. Disruption of this barrier, often referred to as “leaky gut,” is associated with increased intestinal permeability, translocation of luminal antigens and pathogens, and activation of inflammatory responses [12,17]. Probiotics have been shown to strengthen intestinal barrier function through several complementary mechanisms.

Certain probiotic strains, particularly those belonging to the genera *Lactobacillus* and *Bifidobacterium*, can upregulate the expression of tight junction proteins such as occludin, claudins, and zonula occludens (ZO)-1, thereby reducing paracellular permeability [17,18]. In a chick model of *S. typhimurium* infection, *Lacticaseibacillus rhamnosus* P118 strain pretreatment increased the expression of tight junction proteins ZO-1 and Claudin-1, enhanced intestinal mucosal epithelial cell function, and reduced endotoxin and D-lactic acid levels [16]. This effect is mediated in part through the activation of signaling pathways involving protein kinase C (PKC), mitogen-activated protein kinases (MAPKs), and Toll-like receptors (TLRs) [12,17]. Additionally, probiotics stimulate mucus production by goblet cells, enhancing the physical barrier provided by the mucus layer that separates epithelial cells from luminal microbes [18,19]. The probiotic mixture VSL#3, even in the absence of a functional mucus barrier in Muc2-deficient mice, enhances epithelial barrier function by dampening pro-inflammatory cytokine and chemokine production, accelerating tissue restitution, and altering commensal microbiota, with the SCFAs serving as a key mediator [20]. The production of SCFAs, particularly butyrate, serves as an energy source for colonocytes and promotes epithelial cell proliferation and differentiation, further reinforcing barrier integrity [12,17].

### 3.3. Immune Regulation

Probiotics exert profound effects on both innate and adaptive immune responses, contributing to enhanced resistance against infections and reduced inflammatory pathology [9,10,17]. The immunomodulatory properties of probiotics are strain-specific and depend on interactions between microbial surface molecules, such as lipoteichoic acids, peptidoglycans, and flagellin, and host pattern recognition receptors (PRRs), including TLRs and nucleotide-binding oligomerization domain-like receptors (NLRs) [12,18].

In the context of innate immunity, probiotics can enhance the activity of phagocytic cells, natural killer (NK) cells, and dendritic cells, promoting the clearance of invading pathogens [9,10]. They also modulate cytokine and chemokine production, shifting the balance toward anti-inflammatory profiles. For example, *Lactobacillus* and *Bifidobacterium* strains have been shown to induce regulatory T cell (Treg) responses and increase the production of IL-10 and transforming growth factor-beta (TGF-β), while suppressing pro-inflammatory cytokines such as TNF-α and IL-6 [17,18].

In terms of adaptive immunity, probiotics can enhance humoral immune responses by increasing the production of secretory immunoglobulin A (sIgA) in the intestinal mucosa, which plays a crucial role in neutralizing pathogens and preventing their attachment to epithelial surfaces [17,23]. Systemic immune effects have also been observed, including elevated serum levels of immunoglobulins (IgA, IgG, IgM) and enhanced vaccine responses in probiotic-supplemented animals [10,21]. These immunomodulatory effects contribute to improved disease resistance and reduced reliance on antimicrobial treatments in livestock production systems [26,27].

### 3.4. Regulation of Microbial Metabolites

The gut microbiota produces a vast array of metabolites that influence host physiology, metabolism, and immune function. Probiotics can modulate the composition and metabolic activity of the resident microbial community, leading to favorable shifts in metabolite profiles [12,17,28]. Among the most extensively studied metabolites are SCFAs, which are produced through bacterial fermentation of dietary fibers and resistant starches [12,17].

The SCFAs serve multiple beneficial functions in the host. Butyrate is the preferred energy source for colonocytes and promotes epithelial barrier integrity, while acetate and propionate are absorbed into the circulation and influence systemic metabolism [12,17]. Propionate has been shown to suppress hepatic lipogenesis through inhibition of sterol regulatory element-binding protein 1c (SREBP-1c), and both propionate and butyrate activate G-protein-coupled receptors (GPR41 and GPR43) on enteroendocrine cells, stimulating the release of glucagon-like peptide-1 (GLP-1) and peptide YY (PYY), which regulate appetite and glucose homeostasis [12]. *C. butyricum* CBM 588 significantly increased butyrate concentrations in the ceca of broiler chickens, providing energy to enterocytes and contributing to enhanced weight gains [22]. *Lactobacillus acidophilus* D2/CSL supplementation in drinking water increased the abundance of butyrate-producing *Clostridium* species, including *C. asparagiforme*, *C. hathewayi*, and *C. saccharolyticum*, thereby supporting chicken health [24]. In chickens fed *P. pentosaceus* isolated from the mucous membrane of various intestinal segments, higher average SCFA concentrations were observed in the cecum, and these changes were associated with an enrichment of characteristic flavor compounds in breast meat, including (E)-2-heptenal and (E,E)-2,4-nonadienal [23].

Probiotics also influence bile acid metabolism. Primary bile acids synthesized in the liver are conjugated and secreted into the intestine, where they are deconjugated and transformed into secondary bile acids by the gut microbiota [12]. Secondary bile acids activate the farnesoid X receptor (FXR) and the G-protein-coupled bile acid receptor (TGR5), which regulate lipid metabolism, glucose homeostasis, and energy expenditure [12]. Certain probiotic strains, particularly those with BSH activity, can modulate the bile acid pool composition and signaling, with implications for host metabolic health [12,17].

Other microbial metabolites influenced by probiotics include branched-chain amino acids (BCAAs), which have been linked to adipogenesis through mechanistic target of rapamycin complex 1 (mTORC1)-peroxisome proliferator-activated receptor gamma (PPARγ) signaling, and lipopolysaccharide (LPS), a pro-inflammatory component of Gram-negative bacterial cell walls that can trigger TLR4-mediated inflammation and insulin resistance when present at elevated levels due to dysbiosis and increased intestinal permeability [12,25].

## 4. Application of Probiotics in Stress Challenges

Modern poultry production exposes birds to a wide range of environmental stressors that compromise health, welfare, and productivity. Heat stress, breeding environment, mycotoxin contamination, heavy metal exposure, high-altitude hypoxia, and immune challenge each impose distinct physiological burdens. Probiotics have emerged as a dietary strategy to mitigate these stressors by modulating the gut microbiome, reinforcing intestinal barrier integrity, regulating immune and inflammatory responses, and maintaining metabolic homeostasis. The mechanisms and efficacy of probiotic interventions vary considerably across stressor types, administration protocols, and environmental contexts (Table 2).

### 4.1. Heat Stress

Heat stress causes massive economic losses in poultry farming, especially in warm climate zones, impairing feed intake, growth, intestinal structure and immunity via multiple cascading pathways. *B. subtilis* are the most widely validated candidate to counteract thermal damage. Under sustained 30 °C heat exposure for 14 days, dietary *B. subtilis* PB6 elevated weight gain and feed efficiency, boosted circulating growth hormone and IGF-1, improved small intestinal villus morphology, and enriched commensal beneficial bacteria [30]. It stabilized serum glucose and cholesterol, as well as optimized carcass traits by enlarging skeletal muscle and lowering abdominal fat deposition, proving its regulatory role in systemic metabolism beyond simple growth promotion [30].

Under daily cyclic heat stress (32 °C, 10 h/d), continuous *B. subtilis* (1 × 10^6^ CFU/g feed) feeding alleviated heat-related abnormal behaviors (panting, wing spreading, inactivity) and restored feeding and movement behaviors [29]. The heterophil-to-lymphocyte ratio, a canonical stress biomarker, was markedly lowered in probiotic-treated heat-stressed broilers. At the molecular level, *B. subtilis* suppressed hepatic pro-inflammatory IL-6 and HSP70 while elevating anti-inflammatory IL-10 to balance the inflammatory response; reduced cecal sIgA and IgY further implied the probiotic relieved overactivated mucosal immunity triggered by heat stimulation [29].

Combined additives produce synergistic protection against heat stress. Co-supplementation of *S. cerevisiae* and *L. acidophilus* with vitamin E/C achieved better production performance than single probiotic or vitamin treatment alone [31]. Likewise, mixed probiotics, combining *L. acidophilus* (2 × 10^10^ CFU/g) and *B. subtilis* (1.8 × 10^9^ CFU/g) with clove essential oil, simultaneously improved growth, nutrient digestibility and digestive enzyme activity, suppressed TNF-α and lipid peroxidation (lower MDA), enhanced antioxidant enzyme activity (SOD, GSH-Px), enriched intestinal *Lactobacillus* and inhibited pathogenic *E. coli* [32]. Such formulas jointly regulate inflammation, redox homeostasis and gut microbiota to mitigate heat damage.

Heat stress severely disrupts intestinal barrier function. Under cyclic mild heat stress (29.3 ± 2.6 °C), *B. subtilis* (DSM 32315) still improved daily weight gain despite universally reduced feed intake in heat-challenged flocks [33]. It persistently upregulated claudin-5 expression and optimized villus-crypt structure, yet tight junction proteins displayed divergent responses: ZO-1 rose under thermoneutral conditions, while occludin was suppressed under heat, revealing strain-dependent protective effects that differ across tight junction subtypes and environmental contexts [33].

Current heat stress probiotic research is limited by heavy focus on *B. subtilis* with few cross-strain comparisons, simplified short-term lab thermal models that poorly mirror farm compound stress, and fragmented mechanistic data unable to clarify core causal pathways. Though single-strain *Bacillus* powder and liquid lactic acid probiotics each have unique strengths, plus synergies with antioxidants/plant oils, standardized application protocols and cost evaluations are missing.

### 4.2. Breeding Environment

Suboptimal housing conditions, including high stocking density, reused litter, and poor sanitation, create multifactorial environmental challenges distinct from controlled heat stress models. The efficacy of probiotics under such conditions has yielded mixed results. Broilers raised under higher environmental challenge, characterized by reused wood shavings litter, high bird density (16 birds/m^2^), and less frequent drinker cleaning, showed decreased feed intake, weight gain, and drumstick and thigh yield, along with increased intestinal crypt depth [34]. However, supplementation with a multistrain probiotic containing *L. acidophilus* (1 × 10^9^ CFU/g), *B. subtilis* (2.8 × 10^9^ CFU/g), *Bifidobacterium bifidum* (2 × 10^9^ CFU/g), and *E. faecium* (2 × 10^9^ CFU/g) did not significantly improve performance, slaughter variables, or carcass composition regardless of environmental challenge level [34]. Birds receiving the probiotic actually had deeper intestinal crypts, which may indicate increased cell turnover rather than improved gut health [34]. These results caution against assuming universal probiotic efficacy across all environmental contexts and highlight the importance of matching probiotic strains and doses to specific stress conditions.

In contrast, novel *Bacillus* strains have shown promise under suboptimal rearing conditions. Broilers raised under standard commercial conditions but without antibiotic growth promoters and supplemented with *B. pumilus* or *B. subtilis* at appropriate doses exhibited increased body weight and feed intake compared to unsupplemented controls [35]. The high-dose *B. subtilis* group showed elevated CD4^+^CD8^−^CD25^+^ regulatory T cells expressing high levels of interleukin-10 and interferon-gamma in cecal tonsils, increased cecal *Lactobacillus* and *Clostridium* populations, and enhanced ileal expression of tight junction proteins including occludin, zona occludens-1, and junctional adhesion molecule-2 [35]. Mucin-2 and interleukin-17F expression remained elevated through day 42 in the *B. subtilis* groups, indicating sustained intestinal barrier reinforcement and immune modulation [35]. These findings suggest that carefully selected *Bacillus* strains can compensate for the absence of antibiotic growth promoters under standard production conditions.

High-altitude environments impose unique physiological stressors, including hypoxia and increased susceptibility to ascites and coccidiosis. Probiotic supplementation in broilers reared at high altitude did not significantly improve weight gain, feed intake, feed conversion ratio, or water intake [44]. However, mortality from ascites and coccidiosis was reduced in probiotic-treated groups, suggesting that the primary benefit under hypoxic conditions may be improved survivability rather than enhanced growth performance [44]. This distinction is important for production systems where mortality reduction is a critical economic and welfare concern.

### 4.3. Mycotoxin

Aflatoxin B_1_ and zearalenone are widespread mycotoxin pollutants in poultry feed that impair growth performance, disrupt intestinal microbiota and damage visceral organs. Compound probiotics blended with *B. subtilis* (CGMCC 1.0504), *L. casei* (CGMCC 1.2884) and *C. utilis* (CGMCC 2.0615) at optimized proportions mitigate composite mycotoxin toxicity via two distinct, synergistic pathways: direct mycotoxin degradation and indirect gut microbiota regulation [36].

Probiotics produce intracellular hydrolase enzymes to break down toxic mycotoxin structures. Meanwhile, polysaccharides and proteins on bacterial cell walls physically adsorb luminal aflatoxin and zearalenone, cutting toxin absorption across intestinal epithelia. The probiotic intervention containing 4.5 × 10^10^ Lactobacillus cells/kg, including *L. reuteri* (LOCK 1092), *L. plantarum* (LOCK 0860), *L. pentosus* (LOCK 1094), *L. rhamnosus* (LOCK 1091), *L. paracasei* (LOCK 1091), and 4 × 10^6^ *S. cerevisiae* (LOCK 0119) yeast cells per kg feed, reduced hepatic aflatoxin B_1_ residues from 8.9 to 3.7 µg/kg and renal residues from 7.9 to 2.5 µg/kg under low-level aflatoxin contamination. Comparable toxin-clearing effects were also detected in high-toxin diets [37]. Accelerated toxin excretion further alleviated pathological lesions in the liver and kidney [37].

Combined mycotoxin exposure disturbs jejunal microbial homeostasis and elevates overall microbial diversity, while compound probiotics reverse this dysbiosis by enriching beneficial Firmicutes and *Lactobacillus aviarius*. Correlation analysis confirms *L. aviarius* abundance positively correlates with daily weight gain; conversely, tissue mycotoxin residue levels rise with the proliferation of harmful intestinal taxa [36]. Restored balanced microbiota strengthens intestinal barrier function and enhances hepatic detoxification metabolism, indirectly counteracting mycotoxin-induced growth suppression and organ injury.

### 4.4. Heavy Metal

Heavy-metal contamination constitutes a hidden hazard for intensive poultry production. Probiotics mitigate heavy-metal injury by enhancing gastrointestinal sequestration, mediating gut-localized detoxification, and regulating metal-transporter protein expression to reduce intestinal metal absorption and preserve gut-barrier integrity [41]. Lactic acid bacteria with high metal-binding affinity serve as promising candidates for heavy-metal detoxification [40].

Elazab et al. [38] evaluated the protective potential of cinnamon combined with a multi-probiotic blend (*L. acidophilus*, *L. plantarum*, *L. casei*, *L. bulgaricus*, *Streptococcus thermophilus*, *E. faecium*) against copper-sulfate-induced nephrotoxicity in broilers. Relative to copper-exposed controls, this combined intervention lowered copper accumulation in kidney tissue, reduced serum urea, creatinine, and uric acid concentrations, and alleviated renal lipid peroxidation while boosting antioxidant enzyme activity. At the molecular level, the treatment downregulated mRNA of pro-inflammatory mediators (*TNF-α*, *IL-2*, *COX-2*) and pro-apoptotic *Bax*, and upregulated anti-inflammatory *IL-10* and anti-apoptotic *Bcl-2*, confirming robust renal-protective effects against copper toxicity [38]. Cadmium exposure in broilers triggers jejunal villus injury, inflammatory cell infiltration, and suppressed expression of intestinal-barrier-associated genes including *ZO-1*, *ZO-2*, *ZO-3*, *claudin-1*, *claudin-3*, *claudin-4*, *occludin*, and *E-cadherin*. Compound probiotic supplementation effectively rescued these phenotypes, restoring both mRNA and protein abundance of core tight-junction proteins and reshaping inflammatory cytokine profiles [39]. Overall, research on probiotic-mediated heavy metal detoxification in poultry remains very scarce. Meanwhile, the detoxification mechanisms of probiotics have not been directly validated in poultry models; most inferences are extrapolated from rodent and mammal studies, and the strain specificity, dose–response relationship and long-term safety remain to be systematically investigated.

### 4.5. Immune Stress

Immune stress induced by lipopolysaccharide challenge serves as a model for bacterial infection-related stress. Supplementation with *B. coagulans* or *L. plantarum* in broilers subsequently challenged with *E. coli* lipopolysaccharide improved growth performance during the pre-challenge period and modulated the inflammatory response after challenge [43]. Both probiotics reduced serum endotoxin, diamine oxidase, and D-lactic acid levels, indicating improved intestinal barrier integrity [43]. Immunoglobulin Y, IgA, and IgM concentrations increased in the *B. coagulans* (5 × 10^9^ CFU/kg) group, while pro-inflammatory cytokine and interferon-β levels decreased and anti-inflammatory cytokine levels increased in both probiotic groups [43]. *B. coagulans* additionally enhanced antioxidant capacity by increasing glutathione peroxidase, superoxide dismutase, and catalase activities while reducing malondialdehyde levels [43]. Cecal microbiota analysis showed increased *Ruminococcaceae* and reduced *Desulfovibrio* abundance in both probiotic groups, with *B. coagulans* particularly enriching beneficial bacterial populations [43].

Behavioral responses to immune stress are also modifiable by probiotic combinations. Broilers supplemented with a combination of probiotics and egg yolk immunoglobulin Y showed improved standing frequency and latency approach score to a novel object, indicating reduced fearfulness and enhanced welfare [42]. After lipopolysaccharide challenge, this combination group achieved the best gait score and showed lower heterophil-to-lymphocyte ratios, heterophil differential counts, and eosinophil differential counts compared to unsupplemented challenged birds [42]. The combined supplementation appeared to buffer the behavioral and physiological consequences of immune activation more effectively than either component alone.

## 5. Probiotics as Alternatives to Antibiotics

The global poultry industry has relied heavily on in-feed antibiotics for decades to improve feed efficiency, prevent disease, and enhance growth performance [3,45]. However, the emergence of antibiotic-resistant pathogens, the destruction of beneficial gut bacteria, and consumer demand for medication-free poultry products have driven an urgent search for effective alternatives [45,46]. Probiotics’ capacity to control pathogenic bacteria, prevent infectious diseases, and reduce mortality without contributing to antimicrobial resistance makes them particularly attractive for sustainable poultry production [45,46]. We sorted representative functional strains, core regulatory phenotypes, and comparative efficacy against challenged or antibiotic-treated groups into Table 3 for intuitive comparison of strain targeting characteristics and application potential.

### 5.1. Necrotic Enteritis

Necrotic enteritis (NE) is a devastating disease caused by *C. perfringens* that costs the poultry industry billions of dollars annually. *C. butyricum* (HJCB 998) supplementation in broilers challenged with *E. coli* K88 promoted immune responses, improved intestinal barrier function, and enhanced digestive enzyme activities, with no significant differences observed between the probiotic treatment and colistin sulfate antibiotic treatment, suggesting that *C. butyricum* could serve as a viable antibiotic alternative [47]. In a subclinical NE model, *L. johnsonii* BS15 prevented growth decline by enhancing intestinal mucosal immunity in the ileum, inhibiting the NE-caused decrease in immunoglobulins, controlling T-cell subset alterations, and reducing mitochondrion-mediated apoptosis [48]. The probiotic also improved antioxidant abilities in the ileum and maintained villus integrity despite the infection [48]. *B. licheniformis* H2 (CCTCC M2011133) supplementation in NE-challenged broilers improved feed conversion ratio and serum antioxidant status while upregulating catabolism-related genes such as peroxisome proliferator-activated receptor-α and carnitine palmitoyltransferase-1 in the liver, indicating that the probiotic enhanced growth through modulation of lipid metabolism and oxidative stress pathways [49].

A novel multistrain *Bacillus* probiotic containing 4 *B. subtilis* (CPB 011, CPB 029, HP 1.6, and D 014) and 2 *B./velezensis* (CBP 020 and CPB 035) strains evaluated in broilers challenged with *C. perfringens* type A achieved higher final body weights and improved feed conversion ratios compared to the unsupplemented negative control, with increased duodenal villi height and villus height-to-crypt depth ratio in the duodenum and jejunum [50]. *Bacillus* also significantly reduced relative liver weights, indicating improved gut health and reduced bacterial translocation [50]. Similarly, a *Bacillus* direct-fed microbial tested in a laboratory NE challenge model significantly improved body weight, reduced lesion scores, decreased intestinal IgA and fluorescein isothiocyanate-dextran levels, and modulated the ileal microbiota by reducing *Proteobacteria*, *Clostridium*, *Turicibacter*, *Enterococcus*, and *Streptococcus* while increasing *Lactobacillus* and *Bacillus* [51]. The microbial community structure in probiotic-fed birds showed no significant differences from the unchallenged negative control, suggesting that *Bacillus* restored gut homeostasis disrupted by NE [51].

*E. faecium* (NCIMB 11181) pretreatment ameliorated NE-induced intestinal barrier injury by upregulating Claudin-1 gene and protein expression, increasing HSP70 protein levels, and modulating intestinal mucosal immune responses through MyD88, NF-κB, iNOS, PI3K, GLP-2, IL-1β, IL-4, and HSP70 mRNA expression [52]. The probiotic also improved intestinal microbial composition regardless of NE treatment [52]. Claudin-3 is a core component of intestinal tight junctions, and its upregulation is usually regarded as a compensatory response to increased intestinal permeability and bacterial translocation. The downregulation of Claudin-3 mRNA in the *B. licheniformis* group, together with the reduced intestinal lesion scores, indicates that the probiotic alleviates *C. perfringens*-induced epithelial damage and inflammatory response, thus reducing the compensatory overexpression of tight junction proteins [53]. Meanwhile, upregulated MUC2 expression reflects enhanced mucus secretion by goblet cells, which strengthens the physical mucus barrier and blocks pathogen invasion [53]. The probiotic upregulated mRNA levels of TRIF, NF-κB, TLR-4, growth factors (GLP-2 and TGF-β2), and heat shock proteins (HSP60, HSP70, and HSP90), while enriching *Lachnospiraceae_UCG_010* in the gut microbiota [54]. *C. butyricum* YH 018 inhibited the increase of IL-17A gene expression and reduction of Claudin-1 expression caused by *C. perfringens* challenge, while increasing anti-inflammatory IL-10 expression [54]. Although the probiotic decreased the abundance of *C. perfringens* in the gut, it could not significantly affect the occurrence of intestinal lesions or fully correct the shift in gut bacterial composition post-infection, indicating that more effective supplementation schemes may be needed [54].

Several studies have directly compared probiotics with antibiotics in NE models. A comparative evaluation of a postbiotic (nonviable *Lactobacillus* fermentation), a probiotic (*B. subtilis* and *B. licheniformis* mixture), and amoxicillin against NE showed that the combined feed and water postbiotic treatment produced the most promising results, with mortality rates of 22%, 10%, 16%, 22%, 12%, and 20% in the postbiotic dry, postbiotic plus antibiotic, postbiotic dry and aqueous, probiotic dry, probiotic plus antibiotic, and probiotic dry and aqueous groups, respectively, compared to 36% in the untreated challenged group [55]. The best feed conversion ratio was detected in the postbiotic dry and aqueous group (1.51), and the European production efficiency factor was significantly improved in this group (279.33) compared to the untreated challenged group (177.33) [55]. In a naturally occurring NE model, probiotic supplementation reduced mortality, improved feed conversion ratio, and significantly reduced lesion scores in the duodenum and jejunum compared to the negative control, with effects comparable to the antibiotic virginiamycin [56]. The probiotic increased expression of claudin-3, IL-10, IL-17, AMPK-α1, and SGLT1 mRNA, indicating improvements in tight junction integrity, immune modulation, and nutrient transport [56]. *B. subtilis* (DSM 32315) supplementation in *C. perfringens*-infected broilers reduced the severity of NE lesions, improved feed efficiency on day 28, and enhanced body weight gain and feed conversion ratio on day 42, while the diversity, composition, and predictive function of the intestinal microbiota changed with infection, but probiotic supplementation reduced these variations [57].

*L. plantarum* 16 and *Paenibacillus polymyxa* 10 both ameliorated intestinal structure damage caused by *C. perfringens* infection, reversed the apoptosis induced by increased Bax and p53 expression and decreased Bcl-2 expression, and reduced inflammation evidenced by lower levels of IFN-γ, IL-6, IL-1β, iNOS, and IL-10 in the ileum mucosa [58]. Both probiotics restored the intestinal microbiota imbalance by reducing *Firmicutes* and *Proteobacteria* and increasing *Bacteroidetes* at the phylum level and reversed metabolic pathways including B-vitamin biosynthesis, peptidoglycan biosynthesis, and pyruvate fermentation to acetate and lactate [58]. *B. subtilis* (DSM 29784) alleviated intestinal lesions, increased the villus height-to-crypt depth ratio, enhanced maltase activity in the duodenum, and decreased caspase-3 protein expression in the jejunum mucosa of SNE broilers [59]. The probiotic increased the abundance of beneficial *Ruminococcaceae* and *Bifidobacterium* and altered the gut metabolome [59]. *Limosilactobacillus fermentum* (CGMCC 1.2029) and *B. coagulans* both attenuated intestinal inflammation and microbial dysbiosis soon after *C. perfringens* challenge, with *L. fermentum* conversely modulating the increase in *Romboutsia* spp. and the decrease in *Lactobacillus* spp. in the ileum [60]. *B. licheniformis* H2 feeding significantly reduced the relative abundance of *C. perfringens* in the ileum, ameliorated pathological damage in the ileum and liver, enhanced intestinal barrier function and epithelial renewal, reduced energy consumption, and improved enteral nutrition absorption in subclinical NE-infected broilers [61].

### 5.2. Salmonella

The application of probiotics for *Salmonella* control has also been extensively investigated. *S. typhimurium* infection causes dysbiosis by decreasing the abundance of beneficial microbial genera such as *Blautia*, *Faecalibacterium*, and *Lactobacillus*, while increasing genera such as *Escherichia*, *Shigella* and *Flavonifractor* [63]. Continuous feeding of a *Bacillus*-based probiotic restored the gut microbial balance, increased propionate levels, decreased the overall mean load of *Salmonella* in feces, and had a significant effect on *Salmonella* load reduction in internal organs [63]. *B. subtilis* and organic acid blends showed similar effects to the antibiotic avilamycin in broilers exposed to *S. typhimurium* during the starter phase, with no significant differences in body weight, feed conversion ratio, or production efficiency factor between the treatments [65]. *E. faecium* (NCIMB 11181) pretreatment attenuated *S. typhimurium*-induced gut injury by blocking *Salmonella* intestinal colonization and translocation, attenuating gut morphological structure damage and intestinal cell apoptosis and promoting the production of anti-*Salmonella* antibodies in intestinal mucosa and serum [64]. The probiotic also ameliorated gut microbial dysbiosis by enriching *Lachnospiracease* and *Alistipes* levels and suppressing *Barnesiella* abundance [64].

*L. salivarius* 59 and *E. faecium* PXN-33 combined administration achieved a statistically significant 2-log reduction of *S. enteritidis* colonization in the ileum, cecum, and colon at day 43, whereas no reduction was observed when either probiotic was administered individually [66]. The mechanism of inhibition was attributed to a reduction in pH by the probiotic bacteria [66]. *L. rhamnosus* GG supplementation significantly reduced *S. typhimurium* load in chickens raised on built-up litter floor, with 5.95-log and 3.74-log reductions at 7 and 14 days post-infection, respectively [67]. Gut microbiota analysis revealed increased abundance of beneficial genera such as *Butyricicoccus*, *Erysipelatoclostridium*, *Flavonifractor*, and *Bacillus* in LGG-treated groups, and histomorphometry analysis demonstrated increased villus height and villus height-to-crypt depth ratio in the ileum [67]. *L. plantarum* supplementation protected yellow-feathered broilers from *S. typhimurium* infection by easing weight loss post-infection, lowering bacterial counts in the liver, spleen, and cecum, and attenuating liver pathological damage [68]. The probiotic fended off *Salmonella* invasion by affecting the bacterial population of *Anaerotruncus*, *Colidextribacter*, and *Lactobacillus* [68].

In laying hens, *B. subtilis* and *Bacillus methylotrophicus* supplementation in *Salmonella gallinarum*-challenged birds improved egg production post-challenge, reduced *Salmonella* counts in excreta, and increased *Lactobacillus* counts in the excreta [69]. The *Salmonella* counts in the small and large intestine tended to be lower in probiotic-supplemented groups compared to the positive control [69]. *L. fermentum* strains PC-10 and PC-76 significantly decreased the growth of *Salmonella gallinarum* (3.92 ± 0.37 and 3.99 ± 0.22 log_10_ CFU/g) compared to the positive control group (6.88 ± 0.2 log_10_ CFU/g) on day 35, with increased *Lactobacillus* counts and reduced coliform counts [62]. Probiotic-fed groups exhibited less lesion scores and mortality rates, higher weight gain, and improved immune response to Newcastle disease and infectious bursal disease vaccines [62].

### 5.3. Campylobacter jejuni

For *C. jejuni* control, *B. pullicaecorum* 25-3^T^ (LMG 24109) supplementation significantly lowered the abundance of *Campylobacter* spp. in the cecum and *Enterococcus*, *Escherichia*, *and Shigella* spp. in the ileum at day 40 and contributed to the prevention of necrotic enteritis in broilers [46]. The probiotic also decreased the feed conversion ratio significantly (1.518 vs. 1.632) [46]. A wide variety of feed additives tested against *C. jejuni* showed that at 35 days of age, three treatments still had a significant effect, with a maximum reduction of 1.88 log_10_ CFU/g for a probiotic, and at 42 days, a probiotic and a prebiotic-like compound significantly decreased contamination by a maximum of 3 log_10_ CFU/g [75]. *L. salivarius* SMXD51 treatment in broilers artificially contaminated with *C. jejuni* showed a significant reduction of 0.82 log at day 14 and a highly significant reduction of 2.81 log at day 35, with 73% of chickens treated with the culture exhibiting *Campylobacter* loads below 7 log_10_ CFU/g [73]. The probiotic partially limited the impact of *Campylobacter* on the decrease of *Anaerotruncus* spp. and *Subdoligranulum* spp. increase [73]. A multispecies probiotic [*Lactococcus lactis* (IBB 500), *Carnobacterium divergens* S-1, *L. casei* (LOCK 0915), *L. plantarum* (LOCK 0862), and *S. cerevisiae* (LOCK 0141)] added to feed in a commercial farm setting reduced the extent of *Campylobacter* spp. invasion in the gastrointestinal tract, diminished contamination levels in the bird environment, and contributed to improved hygienic parameters of poultry carcasses [72]. *L. johnsonii* FI9785 administered prophylactically led to a reduction in *C. jejuni* colonization in cecal contents. However, the biocontrol seemed reliant upon a high level of initial colonization by the probiotic [71]. Coadministration of a *C. jejuni* N-glycan-based vaccine with *Anaerosporobacter mobilis* (DSM 15930) or *L. reuteri* CSF8 resulted in increased vaccine efficacy, antibody responses, and weight gain in broiler chickens [70]. Synbiotic administration with *Bifidobacterium longum* PCB133 and xylo-oligosaccharide starting from the first day of life was more successful in reducing *C. jejuni* and *Campylobacter* spp. when administered lifelong compared to shorter supplementation [74].

### 5.4. Escherichia coli

The control of *E. coli* infections has also been addressed with probiotic strategies. *E. faecium* NCIMB11181 supplementation in *E. coli* O78-challenged broilers significantly increased body weight, improved average daily gain, dramatically declined death rate, improved jejunal villus height and villus height-to-crypt depth ratio, and upregulated claudin-1 and Mucin2 mRNA expression [76]. The probiotic upregulated PPAR-γ and IL-10 mRNA expression and downregulated NF-κB, TLR4, and IL-1β in the spleen, while the increased liver *E. coli* number caused by the challenge was significantly reversed [76]. *B. subtilis* (Calsporin^®^, Calpis Co. Ltd. Japan) supplementation in broilers intramuscularly inoculated with *E. coli* increased body weight, decreased feed conversion ratio, improved vaccine titers for Newcastle disease, infectious bronchitis, avian influenza, and infectious bursal disease, and reduced *E. coli*, coliform, and *Salmonella* populations in the ceca [77]. Both *B. subtilis* and bacitracin methylene disalicylate increased nutrient digestibility, although the growth reduction caused by *E. coli* challenge could not be fully compensated [77]. A commercial multistrain probiotic blend, consisting of *B. subtilis* MORI 91 (2 × 10^8^ CFU/g), *C. butyricum* M7 (2.06 × 10^8^ CFU/g), *L. plantarum* K34 (2 × 10^8^ CFU/g), functional organic acids, soluble β-glucan, nucleotides, and deoxynojirimycin, significantly enhanced weekly body weight, elevated total cecal *Lactobacillus* populations, and reduced mortality as well as *Escherichia coli* recovery rates in the liver and spleen of broilers challenged with avian pathogenic *E. coli* O78 [78]. Probiotic supplementation also significantly increased hot carcass weight [78].

### 5.5. Coccidiosis

Coccidiosis, a protozoan disease causing severe intestinal destruction, has also been targeted with probiotic interventions. The combination of probiotics and coccidiosis vaccine resulted in an enhanced protective effect against an *Eimeria* challenge, with vaccinated-plus-probiotic birds exhibiting lower lesion scores in the duodenum and higher weight gains compared to birds receiving the vaccine alone [79]. *B. subtilis* probiotic feeding in the presence of *Eimeria tenella* infection increased the abundance of commensal genera including *Clostridium sensu stricto 1*, *Corynebacterium*, *Enterococcus*, *Romboutsia*, *Subdoligranulum*, *Bacillus*, *Turicibacter*, and *Weissella*, which have roles in butyrate production, anti-inflammation, and modulation of protective pathways against pathogens [80]. Dietary supplementation with several *Bacillus* strains in chickens challenged with mixed coccidia infection (*E. tenella*, *Eimeria maxima*, and *Eimeria acervulina*) showed that three strains significantly improved relative body weight gain (95–100% vs. 70% in controls), lowered lesion scores (0.8–0.9 vs. 1.4–2), and reduced total fecal oocyst counts by 10–20 folds [81]. The *Bacillus*-fed chickens showed significant pro- and anti-inflammatory responses, higher expression of antioxidants (SOD1 and HMOX1), and tight junction proteins (JAM2 and occludin) in the ceca, duodenum, and jejunum [81]. Probiotic and *Bidens pilosa* supplementation (individually or in combination) appeared effective in inhibiting the occurrence of coccidiosis and decreasing disease severity, with reduced oocyst shedding, caecal lesion scores, and mortality, while enhancing antioxidant enzymes, pro-inflammatory cytokines, and tight junction protein expression [82]. Probiotics and prebiotics administered with live coccidia vaccines did not significantly reverse the reduction in growth performance during the first three weeks of age but continued to exhibit protection levels comparable to the vaccine alone following homologous coccidia challenge [83].

## 6. Early-Life Intervention and Administration Strategies

The timing and method of probiotic administration critically influence colonization success, microbiota development, and long-term health outcomes in poultry. Because the intestinal microbial community in chickens reaches a near-stable state within the first week of life, the window for permanent microbiota remodeling is narrow [84]. Early colonizers determine the trajectory of community assembly, and once the microbiota matures, stable communities resist further modification [84]. This principle underpins the rationale for early-life intervention strategies, including in ovo delivery, at-hatch inoculation, and continuous supplementation through feed or water.

### 6.1. Early-Life Intervention Strategies

#### 6.1.1. In Ovo Delivery

In ovo administration delivers probiotics directly to the developing embryo, allowing beneficial bacteria to colonize the gut before hatch and compete with potential pathogens that are encountered immediately after emergence [85,86]. This approach bypasses the delay in microbial acquisition that occurs in commercial hatchery environments where chicks are hatched in clean conditions without contact with adult hens [87]. The absence of maternal microbiota transfer means that newly hatched chicks lack key bacterial groups such as Bacteroidetes and Actinobacteria, which are normally transferred through hen contact within the first 24 h after hatch [87]. In ovo probiotic delivery can partially compensate for this deficit by introducing beneficial strains at a developmental stage when the gut is still sterile and receptive to colonization [85].

Multiple studies have demonstrated the feasibility and benefits of in ovo probiotic administration. When *B. subtilis* was injected into the amnion of broiler embryos at day 18.5 of incubation, hatch parameters remained largely unaffected, with only the percentage of pipped eggs showing a significant difference compared to non-injected controls [88]. In ovo delivery of *B. subtilis* enhanced intestinal morphology, increasing ileal villus width by 18% and jejunal villus height by 23% compared to saline-injected controls, without compromising hatch performance or gut homeostasis [88]. A subsequent study comparing two doses of *B. subtilis* 10SI delivered in ovo at day 18 found that both doses improved chick body weight relative to egg weight at hatch and reduced feed intake during week 5, although overall body weight gain and feed conversion ratio did not differ from controls [89]. In ovo-delivered *B. subtilis* 10SI also reduced serum IgG levels compared to untreated controls, suggesting that early probiotic exposure may modulate humoral immunity by neutralizing pathogenic organisms that would otherwise stimulate natural antibody production [89].

In ovo administration of a *Bacillus* spp. (2 *B. amyloliquefaciens* and 1 *B. subtilis*)-based probiotic at day 18 of embryogenesis significantly reduced the horizontal transmission of virulent *E. coli* in neonatal broiler chickens [90]. Chicks that received the in ovo probiotic had lower total Gram-negative bacterial counts at the day of hatch and day 7, higher body weight at both time points, and greater body weight gain during the first week [90]. Microbiota analysis revealed that the probiotic treatment reduced the relative abundance of Proteobacteria and Enterobacteriaceae while increasing Firmicutes and *Lachnospiraceae*, along with enrichment of the genus *Oscillospira* [90]. Beta diversity analysis showed a significant difference in bacterial community structure between treated and control groups, indicating that in ovo probiotic administration can alter the trajectory of gut microbial assembly and confer protection against early pathogen exposure [90].

The dose of probiotic delivered in ovo influences the magnitude and duration of effects. When a commercial probiotic product (Primalac W/S) was injected at embryonic day 18 at doses of 10^5^, 10^6^, or 10^7^ CFU per egg, hatchability was not affected at any dose [86]. However, the intermediate dose (10^6^ CFU) produced significantly greater body weight on day 4 and day 6 post-hatch compared to negative controls and the lowest dose [86]. Gene expression analysis in the ileum and cecal tonsils showed that in ovo probiotic supplementation was associated with initial upregulation of inducible nitric oxide synthase on day of hatch, followed by downregulation of Toll-like receptors 2 and 4, inducible nitric oxide synthase, trefoil factor 2, mucin 2, interferon-γ, and interleukins 4 and 13 in both tissues [86]. These expression patterns differed according to treatment dose, tissue type, and sampling time, demonstrating that in ovo probiotic delivery can modulate intestinal immune development in a dose-dependent manner [86].

Synbiotic combinations delivered in ovo offer additional opportunities for early gut colonization. When *L. salivarius* IBB3154 with galactooligosaccharides (GOS) or *L. plantarum* IBB3036 with raffinose family oligosaccharides (RFO) were injected into Cobb broiler embryos on day 12 of incubation, hatchability remained unaffected [91]. Post-hatch analysis at day 21 revealed that both synbiotic treatments increased ileal populations of *Lactobacillus* spp. and *Enterococcus* spp. compared to controls [91]. Gene expression profiling showed that the *L. salivarius*-GOS synbiotic caused significant upregulation of IL-6, IL-18, IL-1β, IFN-γ, and IFN-β in the spleen on day 21 and IL-1β on day 7, while the *L. plantarum*-RFO synbiotic did not elicit similar immune responses in any tissue examined [91]. These divergent effects highlight that in ovo synbiotic formulations must be carefully designed, as the choice of probiotic strain and prebiotic partner determines the immunological outcome [91].

Combining in ovo probiotics with post-hatch oral administration can produce synergistic immune effects. When a *Lactobacillus* spp. (10^6^ or 10^7^ CFU/100 μL/egg; (*L. salivarius* JB/SL-26, *L. johnsonii* JB/SL-39, *L. reuteri* JB/SL-42, and *L. crispatus* JB/SL-44) cocktail was administered in ovo at embryonic day 18 followed by weekly oral gavage through day 28, birds showed enhanced antibody responses to sheep red blood cells and keyhole limpet hemocyanin, increased percentages of CD4^+^ and CD4^+^CD25^+^ T cells in the spleen, and upregulated expression of IFN-α, IFN-β, IL-8, IL-13, and IL-18 in the spleen and IFN-γ, IL-2, IL-6, IL-8, IL-12, and IL-18 in the bursa [92]. Birds that received only in ovo or only oral administration showed weaker responses, indicating that the combination strategy maximizes immune modulation during the critical early post-hatch period [92].

#### 6.1.2. At-Hatch Inoculation

At-hatch administration represents another early intervention window. Inoculation of broiler chicks immediately after hatch with a mix of three *Lactobacillus* spp. (*L. ingluviei*, *L. agilis*, and *L. reuteri*) resulted in significantly higher body weight by day 28 [84]. Although all three strains could colonize when administered alone, the mixed inoculum resulted in colonization only by *L. ingluviei*, suggesting competitive exclusion among the administered strains [84]. Despite the decline in *L. ingluviei* abundance over time, the effects of at-hatch administration on microbiota development and structure remained persistent, with a tendency to promote beneficial taxa and reduce pathogenic taxa [84]. This finding underscores that successful colonization at hatch does not require permanent persistence of the administered strains; rather, early modulation of community assembly can have lasting effects on microbiota composition [84].

The delivery route of probiotics after hatch also influences their efficacy. Supplementation of *L. acidophilus* D2/CSL in drinking water at two concentrations (0.2 g and 0.02 g per bird per day) showed that the main site of action was the crop, particularly in the early stage of chicken life [24]. At 14 days, *L. acidophilus* D2/CSL abundance was significantly higher in the crops of chickens treated with the high dose compared to controls (14.09% vs. 1.74%), while cecal populations did not differ significantly between groups [24]. The high-dose treatment also increased the abundance of butyrate-producing *Clostridium* spp. (*C. asparagiforme*, *C. hathewayi*, and *C. saccharolyticum*) in the ceca at 35 days, indicating that water-borne probiotics can exert distal effects through metabolic cross-feeding [24].

#### 6.1.3. Feeding Routes: Drinking Water, Mixing with Feed, Microcapsule Coating

Supplementation of probiotics in drinking water at 1% inclusion of *Lactobacillus casei*, *L. acidophilus*, or *Bifidobacterium* improved growth performance, carcass traits, immune function, and antioxidant capacity in broiler chickens [93]. Female chicks showed increased body weight and average daily gain, while male chicks had reduced feed intake and feed conversion ratio [93]. Probiotic supplementation increased the weight of lymphoid organs (spleen, bursa of Fabricius, and thymus), elevated immune-related protein concentrations, and enhanced antioxidase and digestive enzyme activities [93]. Microbial analysis revealed reduced counts of *E. coli* and *Salmonella* and increased *Lactobacillus* populations in the probiotic groups [93].

The timing of synbiotic administration relative to pathogen exposure determines the degree of protection against *C. jejuni* colonization. When a synbiotic containing microencapsulated *B. longum* PCB133 and xylo-oligosaccharide was administered to broiler chicks starting from day 1 (lifelong supplementation) or from day 14 (delayed supplementation), lifelong administration was more effective in reducing cecal *C. jejuni* and *Campylobacter* spp. loads [74]. PCR-denaturing gradient gel electrophoresis analysis revealed that early synbiotic supplementation strongly influenced the cecal microbial community profile, whereas the physiological changes in microbial composition that occur during growth were also evident [74]. This study demonstrates that continuous early intervention is superior to delayed or shorter supplementation for pathogen control [74].

The choice of probiotic delivery method must account for practical considerations in commercial poultry production. In-feed supplementation remains the most common approach because it integrates easily into existing feeding systems and ensures continuous exposure [88]. However, in ovo delivery offers the advantage of earlier intervention, before chicks encounter environmental pathogens, while water-based supplementation provides flexibility for treatments that need to be administered intermittently or in response to specific health challenges [88]. Encapsulation technologies can improve the stability and delivery of probiotics; supplementation of encapsulated *L. acidophilus* and *L. lactis* either alone or in combination significantly increased total serum protein, albumin, and globulin and lowered total serum cholesterol, LDL cholesterol, and triglycerides compared to non-encapsulated probiotics or unsupplemented controls [94].

The identification of bacteria that can colonize and persist in the chicken gut after a single early exposure is critical for developing next-generation probiotics. Studies using microbial inocula from healthy adult chicken donors have identified bacteria that are consistently increased in birds exposed to microbial inoculations, and these can be isolated and employed in future research [95]. Competitive assays comparing bacteria from intensively versus extensively raised chickens have shown that some strains are highly adapted to the chicken gut and can be used to design probiotics that establish stable populations after a single early administration [95]. The combination of in ovo or at-hatch delivery with carefully selected strains that are adapted to the chicken gut environment offers the greatest potential for achieving persistent microbiota modulation and long-term health benefits.

### 6.2. Administration Strategies

The simultaneous administration of probiotics with live vaccines can mitigate the negative effects of vaccination on growth performance while enhancing immune protection. When a live *S. enteritidis* vaccine was administered at day 7 via drinking water, the addition of a commercial probiotic or prebiotic reduced mortality, fecal shedding, and re-isolation of *Salmonella* from internal organs following challenge at day 28 [96]. Probiotic coadministration also improved growth performance compared to vaccination alone, suggesting that probiotics can compensate for the growth depression associated with live vaccine administration [96]. Similarly, coadministration of a *C. jejuni* N-glycan-based vaccine with *L. reuteri* or *A. mobilis* increased vaccine efficacy, antibody responses, and weight gain in broiler chickens [70]. Microbiota analysis of vaccine responders identified *A. mobilis* as significantly more abundant in birds that responded to vaccination, and supplementation of this strain with the vaccine improved the proportion of responders [70].

Probiotics can also be combined with antibiotics to enhance efficacy against multidrug-resistant pathogens. In ovo inoculation of probiotics with florfenicol significantly improved growth performance and prevented colonization of multidrug-resistant *S. enteritidis* in the majority of experimental chicks, with the remaining chicks showing very low colonization levels as detected by RT-PCR [97]. This synergistic approach suggests that in ovo delivery of probiotics alongside carefully selected antibiotics may offer a strategy to control resistant pathogens while minimizing the negative effects of antibiotic exposure on the developing microbiota [97].

The use of probiotics in combination with coccidiosis vaccines requires careful consideration, as probiotics may not fully reverse the growth depression associated with vaccination during the first three weeks of life. When broiler chicks were vaccinated against coccidia at day 1 and received a probiotic or prebiotic supplement, the probiotic group showed improved plasma carotenoid levels post-vaccination compared to the vaccine-only group, but neither probiotic nor prebiotic significantly reversed the reduction in body weight gain during the first three weeks [83]. Following homologous coccidia challenge, vaccinated birds that received probiotic or prebiotic continued to exhibit protection levels comparable to those receiving vaccine alone, with improved performance and reduced oocyst shedding and lesion scores [83].

## 7. Novel Probiotics, Postbiotics, Synbiotics, and Multistrain Formulations

The limitations of single-strain probiotic preparations have driven the development of more sophisticated formulations that leverage synergistic interactions among multiple microbial species, combine live bacteria with prebiotic substrates, or utilize non-viable bacterial products to confer health benefits. These advanced approaches, multistrain probiotics, synbiotics, postbiotics, and novel probiotic candidates, offer distinct advantages over conventional single-strain products in terms of stability, efficacy, and mechanistic diversity.

### 7.1. Multistrain Compound Preparation

Multistrain probiotic formulations have been designed to exploit the complementary metabolic and immunomodulatory activities of different bacterial species. A consortium comprising *Lactobacillus brevis* TN9, *L. salivarius* F9/2, and *L. salivarius* TL4/1 enhanced body weight gain by 13% and improved feed conversion ratio (1.50 versus 1.75) compared to control birds [98]. This consortium also increased serum antibody titers against *Salmonella enterica* lipopolysaccharides and upregulated the anti-inflammatory cytokine IL-10 while suppressing the pro-inflammatory cytokines IL-1β and TNF-α [98]. Similarly, a multistrain probiotic containing *L. plantarum*, *L. fermentum*, *P. acidilactici*, *E. faecium*, and *S. cerevisiae* reduced mortality and intestinal *Pasteurella multocida* counts in broilers challenged with fowl cholera, while upregulating anti-inflammatory genes including hypoxia inducible factor 1 alpha (HIF1A) and tumor necrosis factor-stimulated gene-6 (TSG-6) in the intestinal mucosa [99]. The efficacy of multistrain preparations often exceeds that of individual strains: combined administration of *L. salivarius* 59 and *E. faecium* PXN33 produced a statistically significant 2-log reduction of *S. enteritidis* colonization in the ileum, cecum, and colon, whereas neither probiotic alone achieved significant reduction [66]. This synergy may arise from complementary mechanisms, including pH reduction through organic acid production and competitive exclusion at epithelial binding sites [66].

Bacillus-based multistrain formulations have received particular attention due to the spore-forming capacity of these organisms, which confers superior stability during feed processing and gastrointestinal transit. A novel multistrain *Bacillus* probiotic containing *B. subtilis* and *B. amyloliquefaciens* improved feed conversion ratio in broilers fed a reduced metabolizable energy diet and increased duodenal villus height and villus height-to-crypt depth ratio [50]. In a necrotic enteritis model, the combination of *B. subtilis* (1 × 10^7^ CFU/kg) and *B. amyloliquefaciens* (7.42 × 10^7^ CFU/kg) improved zootechnical performance and reduced intestinal lesion scores compared to challenged controls [100]. Comparative evaluation of different *Bacillus* strain combinations revealed that formulations containing *B. amyloliquefaciens* strains and *B. pumilus* produced the greatest improvements in feed conversion ratio and body weight, with in vitro testing confirming high protease and amylase production by these strains [101]. A probiotic complex combining *B. subtilis* (2  ×  10^8^  CFU/g), *C. butyricum* (2  ×  10^6^  CFU/g), and *E. faecalis* (1  ×  10^6^  CFU/g) improved average daily gain and feed intake from day 22 to 42, increased breast muscle yield and thymic index, and reduced fecal *E. coli*, *Salmonella*, and harmful gas emissions including H_2_S and NH_3_ [102]. The recommended inclusion level of 0.05% provided the most consistent benefits across multiple parameters [102].

### 7.2. Synbiotics

The combination of probiotics with prebiotics in synbiotic formulations aims to enhance the survival and colonization of beneficial bacteria by providing specific fermentable substrates. Their unique synergistic advantages are reflected in two core mechanisms: prebiotics provide exclusive carbon sources for target probiotics and help strains resist damage from feed pelleting, gastric acid and bile salts; meanwhile, short-chain fatty acids generated by prebiotic fermentation further strengthen intestinal barrier and immune homeostasis, producing additive benefits unavailable from single probiotic or prebiotic supplementation.

Common prebiotics used in poultry synbiotics can be categorized into three types with distinct advantages and limitations. Synthetic oligosaccharides including GOS and raffinose oligosaccharides show high strain specificity and stable regulatory effects, yet their industrial cost is relatively high. Inulin and oligofructose are low-cost feed additives that can be utilized by most lactic acid bacteria, though excessive supplementation may trigger intestinal flatulence. Plant and whey-derived natural prebiotics cut breeding costs and improve livestock product quality, but their effective component content varies greatly between raw material batches.

A wealth of representative poultry trials covering in ovo intervention, broilers and laying hens have validated the practical application potential of diverse synbiotic combinations. In vitro screening selected *L. salivarius*-GOS and *L. plantarum*-RFO as stable formulas fit for embryonic in ovo delivery. Post-hatch detection on day 21 confirmed that the *L. salivarius*-GOS synbiotic raised ileal *Lactobacillus* and *Enterococcus* counts and upregulated splenic IL-6, IL-18, IL-1β and IFNγ to accelerate immune maturation of young chicks [91]. In ovo *B. subtilis*, raffinose, and synbiotics can upregulate the mRNA expression of intestinal function-related genes and nutrient transporter-related genes [103]. For broilers, the synbiotic composed of *E. faecium* and GOS markedly improved early growth and continuously inhibited cecal *E. coli* proliferation [104]. Inulin is the most widely studied prebiotic material. Combined feeding of *L. plantarum*, *Lactiplantibacillus pentosus* and inulin enriched butyrate-producing *Blautia*, *Faecalibacterium* and *Lachnospiraceae*, elevated fecal butyrate, and lowered the abundance of aminoglycoside, β-lactamase and lincosamide resistance genes at the metagenomic level [105]. The triple formula of *L. plantarum* AKK30, *S. cerevisiae* B18 and 0.5–1.0% inulin outperformed single probiotics in weight gain, feed conversion efficiency, nutrient retention and small intestinal villus development [106]. For local crossbred chickens, dahlia tuber powder as natural inulin mixed with *Lactobacillus* spp. reduced breast fat and cholesterol while optimizing meat color [107]. In laying hen trials, pairing *P. acidilactici* MA 18/5 with dried whey prebiotic-enriched cecal *Olsenella* and *Lactobacillus crispatus* and metagenomics revealed upregulated genes related to short-chain fatty acid and galactose metabolism to boost nutrient utilization and maintain stable egg production [108].

However, most synbiotics lack strain-prebiotic matching based on carbohydrate metabolism, leading to inconsistent field efficacy. Standardized dosage, feeding schedules and economic assessments are scarce, and metabolite-mediated host molecular pathways remain underexplored.

### 7.3. Novel Candidate Probiotic Strains

Novel probiotic candidates beyond traditional lactic acid bacteria have been explored for their unique functional properties. *B. licheniformis* B4, isolated from camel feces, demonstrated phytase, protease, cellulase, and xylanase activity and survived at pH 3.0 and 1% bile salts [109]. Solid-state fermentation of soybean meal with this strain reduced phytic acid by 73.3%, increased crude protein from 44.8% to 54.3%, and decreased neutral detergent fiber by 38.4%, presenting a promising alternative to phytase additives [109]. A combination of *L. reuteri* and *Streptomyces coelicolor* improved body weight gain by 2%, decreased feed consumption by 7%, and improved feed conversion ratio by 6–7% compared to the control diet [110]. Probiotics for mealworm and superworm larvae were formulated using *L. plantarum* KCTC 3099 and *Saccharomyces cerevisiae* KCTC 7928 mixed in a 1:1 ratio, increasing average daily gain and IgG and IgA levels while reducing feed conversion ratio, mortality, and cecal *E. coli* and *Salmonella* contents in chicks orally challenged with these pathogens [111]. When *Chlorella vulgaris* microalgae was combined with the commercial probiotic Probimais^®^ (A-Biomart, São Paulo, Brazil), which comprised *B. subtilis* (3.6 × 10^9^ CFU/g), *B. bifidum* (2.5 × 10^9^ CFU/g), *E. faecium* (2.6 × 10^9^ CFU/g), and *L. sacidophilus* (1.3 × 10^9^ CFU/g), feed conversion was improved and *Bifidobacterium* spp. abundance was increased in birds receiving 1% microalgae in the diet at day 42 [112].

### 7.4. Postbiotics

The concept of postbiotics, non-viable bacterial products or metabolic byproducts that confer health benefits, has emerged as a promising alternative to live probiotics, particularly for applications where viability is difficult to maintain. Postbiotics derived from *L. acidophilus* (NCDC-15) at 0.2% supplementation produced significantly higher body weight (1677.52 g) and better feed conversion ratio (1.75) compared to antibiotic and probiotic groups, with improved villus height, crypt depth, and jejunal antioxidant values [113]. A postbiotic formulation containing non-viable *Lactobacillus* species fermentation products, administered through both feed and water, markedly decreased the severity of necrotic enteritis signs in broilers challenged with *C. perfringen* [55]. The combined feed and water postbiotic treatment achieved a mortality rate of 16% compared to 36% in the untreated challenged group and produced the best feed conversion ratio (1.51) among all treatment groups, including those receiving antibiotics or live probiotic [55]. Kinome analysis of a postbiotic in the context of *C. perfringens* challenge revealed that the postbiotic predominantly affected innate immune responses and appeared immunomodulatory, reducing pro-inflammatory responses and generating a homeostatic-like response during infection [114].

Extracellular vesicles (EVs) produced by probiotic bacteria represent a novel postbiotic modality that mediates host-microbe communication without the need for live bacterial cells. *L. reuteri* BBC3-derived EVs carried DNA, RNA, and bioactive proteins, including glucosyltransferase, serine protease, and elongation factor Tu [115]. Administration of these EVs to broilers attenuated lipopolysaccharide-induced inflammation, reducing mortality and intestinal injury, suppressing pro-inflammatory gene expression (TNF-α, IL-1β, IL-6, IL-17, IL-8), and improving anti-inflammatory gene expression (IL-10, TGF-β) in the jejunum [115]. The EVs could be internalized by chicken macrophages and suppressed NF-κB activity, while both vesicular proteins and nucleic acids were essential for the immunoregulatory effects [115].

Heat-inactivated probiotics represent another category of postbiotic products that combine the safety profile of non-viable preparations with functional benefits. Heat-inactivated compound probiotics containing *B. subtilis* (1 × 10^8^ CFU/g) and *L. acidophilus* BFI (1 × 10^8^ CFU/g) improved feed efficiency in yellow-feathered broilers from day 1 to 63, decreased plasma cholesterol and creatinine contents, and modulated cecal microbiota composition and diversity [116]. The treatment increased the relative abundances of *Barnesiellaceae* and *L. aviarius* at day 21 and reduced *Lachnoclostridium* at day 42 [116]. *S. cerevisiae* fermentation product, a postbiotic derived from yeast fermentation, improved average daily feed intake and average daily gain during the first 14 days and feed conversion ratio over the entire experimental period compared to control and probiotic groups [117]. Birds receiving the fermentation product had significantly lower *E. coli*, enterohemorrhagic *E. coli*, ESBL-producing *Enterobacteriaceae*, and *Salmonella* counts, as well as increased villus height and villus height-to-crypt depth ratio [117].

### 7.5. Compound Functional Additive Combinations

Matching specific *Lactobacillus* strains with phytobiotics also boosts functional metabolite accumulation and suppresses ex vivo survival of ESBL-positive *E. coli* in crop and cecal contents, double-restraining resistant pathogenic bacteria [118]. Fermented herbal-probiotic composites represent another plant-derived compound form: compound probiotics fermented with *Aspergillus niger*-processed Chinese medicinal herbs improve broiler average body weight and daily gain while lowering feed conversion ratio. This composite formulation enhances systemic antioxidant capacity and intestinal mucosal immunity by raising glutathione peroxidase and secretory IgA concentrations; it also lengthens small intestinal villi, upregulates tight junction genes (*occludin*, *ZO-1*), and enriches beneficial cecal taxa including *Olsenella*, *Barnesiella* and *Lactobacillus* to sustain gut homeostasis [119].

The combination of probiotics with phage cocktails represents an innovative strategy that targets both host microbiota modulation and direct pathogen control. Broilers receiving 0.1% phage cocktail plus 0.1% probiotic showed significantly better body weight, body weight gain, and feed conversion ratio compared to the control group [120]. Unique gut microbiota diversity was observed in the combination groups, with significant enrichment of microorganisms associated with short-chain fatty acid production and upregulation of predicted genes related to carbohydrate and amino acid metabolism [120].

## 8. Future Perspectives

Accumulated evidence confirms that probiotics remodel intestinal microbiota, reinforce gut barrier and immunity, mitigate diverse environmental stressors, and serve as viable alternatives to in-feed antibiotics in poultry production [3,4]. However, inconsistent in vivo efficacy and translational barriers remain prominent obstacles limiting large-scale industrial application.

Rational strain screening and matching systems are lacking. Most current probiotic formulas are developed through empirical combination rather than targeted selection based on poultry breed, growth stage and rearing stress [6]. Multi-omics technologies including metagenomics and metabolomics should be adopted to dissect host–strain molecular interactions, screen strains with persistent intestinal colonization and specialized functional traits, and eliminate blind compounding [2,4].

Novel formulations and delivery schemes demand systematic optimization. Synbiotics, postbiotics, and multi-component compound additives deliver synergistic benefits, yet standardized dosages, feeding schedules, and economic assessment data are insufficient [108]. Early-life intervention via in ovo or at-hatch inoculation is a promising strategy for long-term microbiota programming; microencapsulation technology also needs further upgrading to enhance strain tolerance during feed manufacture and gastrointestinal transit [92].

Unified evaluation criteria and industrial quality control specifications are urgently needed. Divergent detection indicators across studies hinder horizontal data comparison. Standardized assessment frameworks covering growth traits, intestinal morphology, immune cytokines and gut metabolites should be constructed. Meanwhile, complete regulatory norms for strain authentication, viable bacterial count and shelf-life stability must be perfected to guarantee consistent product quality [3,4].

Research should shift from single laboratory stress models to complex multi-stress farm simulation. Most existing trials adopt isolated heat, mycotoxin or pathogen challenge, failing to mimic the overlapping stress conditions of commercial flocks [1]. Field validation under actual breeding environments is essential to reduce the deviation between laboratory outcomes and on-farm performance [6].

The public health significance of probiotics requires deeper exploration. Targeted strains capable of lowering poultry carriage of zoonotic pathogens such as *Salmonella* and *Campylobacter* help cut foodborne disease risks and alleviate global antimicrobial resistance crises, which should be prioritized in follow-up research.

## 9. Conclusions

Probiotics are eco-friendly and multifunctional substitutes for poultry antibiotic growth promoters. They reshape gut microbiota, strengthen intestinal barriers and modulate immunity. They also ease various breeding stresses, inhibit bacterial diseases and reduce zoonotic pathogen loads to boost production performance. However, unstable field efficacy limits their industrial use. The main reasons include random strain combinations, non-uniform administration schemes, inconsistent evaluation indicators and oversimplified single-stress lab models. Future research can adopt multi-omics to analyze host–strain–microbe interactions for targeted strain screening. Researchers should systematically optimize synbiotics, multistrain probiotics, postbiotics, and early-intervention delivery technologies. Unified evaluation systems and industrial quality control standards need to be set up. Multi-stress field trials should focus on reducing Salmonella and Campylobacter in poultry to mitigate antimicrobial resistance. In-depth mechanism exploration, rational formula design, standardized delivery and field verification will jointly support stable large-scale application of new probiotic products in sustainable antibiotic-free poultry farming.

## Figures and Tables

**Table 1:** Probiotic mechanisms of action in poultry.

Mechanism Category	Representative Probiotic Strains	Action Pathway	References
Regulation of gut microbiota composition	*Lactobacillus reuteri*, *Pediococcus acidilactici*, *Pediococcus pentosaceus*, *Enterococcus faecium*	Competitive exclusion of pathogenic bacteria	[4,9,10,11]
	Lactic acid bacteria, *Bacillus* spp. (eg., *B. subtilis* KATMIRA1933 and *B. amyloliquefaciens* B-1895)	Production of antimicrobial metabolites (bacteriocins, organic acids)	[9,12,13,14]
	Lactic acid bacteria, *B. subtilis* PS-216	Enrichment of beneficial commensal bacteria (*Lactobacillus*, *Bifidobacterium*)	[11,15]
Maintenance of intestinal barrier integrity	*B. subtilis*, *Clostridium butyricum*, *Lacticaseibacillus rhamnosus* P118	Upregulation of tight junction proteins (occludin, ZO-1, claudin-1)	[16,17,18]
	*Bacillus licheniformis*, *L. rhamnosus*, *Lactiplantibacillus plantarum*	Promotion of intestinal epithelial cell proliferation and villus development	[12,16,17]
	*Enterococcus faecium* NCIMB 11181, VSL#3 probiotic mixture, *B. subtilis*	Enhancement of mucus layer and Muc2 expression	[18,19,20]
Immunomodulation	*B. subtilis*, *L. salivarius*, *Bifidobacterium* spp.	Stimulation of secretory IgA production at mucosal surfaces	[17,18,21]
	*Bacillus coagulans*, multistrain probiotic	Modulation of pro-/anti-inflammatory cytokine balance	[17,18]
	*B. subtilis*, *Enterococcus faecium*, *Lactobacillus* spp.	Activation of TLR signaling and innate immune pathways	[12,18]
Improvement of nutrient metabolism	*C. butyricum* CBM 588, *Butyricicoccus pullicaecorum*	Production of short-chain fatty acids via fermentation	[12,17,22]
	*Bacillus*-based direct-fed microbials, *P. pentosaceus*	Enhancement of digestive enzyme activity and nutrient digestibility	[3,23]
	Synbiotic formulations, *Lactobacillus acidophilus* D2/CSL	Modulation of host metabolic pathways (amino acid, carbohydrate)	[12,24]

**Table 2:** Typical probiotic strains and their core regulatory pathways under common poultry stressors.

Stress Type	Representative Probiotics	Regulatory Mechanism	Efficacy	References
Heat stress	*B. subtilis*; *Saccharomyces cerevisiae* and *L. acidophilus* and vitamin E/C; *B. subtilis* and clove essential oil	Balance pro/anti-inflammatory cytokine profiles; upregulate tight junction proteins; enhance systemic antioxidant capacity	Improve average daily gain and feed efficiency; lower heterophil-to-lymphocyte ratio; alleviate hepatic HSP70 and IL-6 overexpression; optimize carcass composition	[29,30,31,32,33]
Suboptimal breeding environment	*Bacillus pumilus*, *B. subtilis*, *multistrain probiotic (Lactobacillus* spp., *Bifidobacterium bifidum)*	Reinforce intestinal barrier; induce regulatory T cell differentiation; maintain gut microbial homeostasis	Increase body weight and feed intake; upregulate occludin and ZO-1; enrich cecal *Lactobacillus* and *Clostridium* populations	[34,35]
Mycotoxin contamination	Compound probiotics (*B. subtilis*, *Lactococcus casei*, and *Candida utilis*), *Lactobacillus* spp.	Direct mycotoxin adsorption and enzymatic degradation; indirect regulation of jejunal microbiota	Reduce aflatoxin B_1_ residues in liver and kidney; restore microbial richness; alleviate histopathological damage of visceral organs	[36,37]
Heavy metal exposure	Multi-probiotic blend and cinnamon; compound probiotics	Gastrointestinal metal sequestration; modulate metal transporter expression; alleviate renal oxidative injury and apoptosis	Lower copper accumulation in kidney tissue; restore tight junction protein expression; reduce serum urea, creatinine and lipid peroxidation levels	[38,39,40,41]
Immune stress	*B. coagulans*; *L. plantarum*; probiotics and egg yolk IgY	Protect intestinal barrier integrity; shift cytokine balance to anti-inflammatory profile; enhance antioxidant enzyme activity	Reduce serum endotoxin and D-lactic acid; elevate immunoglobulin levels; mitigate stress-induced behavioral abnormalities	[42,43]

**Table 3:** Representative probiotics for the prevention of major poultry bacterial diseases and their core functions.

Target Disease	Representative Probiotics	Action Mechanism	Efficacy	References
Necrotic enteritis	Single strain: *C. butyricum*, *L. johnsonii* BS15, *B. licheniformis* Compound: Multistrain *Bacillus* (*B. subtilis* and *B. amyloliquefaciens*)	Inhibit *C. perfringens* colonization; repair intestinal tight junctions; modulate mucosal immunity and antioxidant status	Improve feed conversion ratio; reduce intestinal lesion scores; restore gut microbial homeostasis; partial regimens achieve efficacy comparable to colistin or virginiamycin	[47,48,49,50,51,52,53,54,55,56,57,58,59,60,61]
Salmonellosis	Single strain: *B. subtilis*, *E. faecium* NCIMB 11181, *L. rhamnosus* GG Compound: *L. salivarius* and *E. faecium*	Competitive exclusion of pathogens; enhance secretory IgA secretion; reshape cecal microbiota	Reduce Salmonella load in visceral organs by up to 3.74 log; achieve 2-log reduction of *S. enteritidis* in the intestine; deliver pathogen inhibition comparable to avilamycin	[62,63,64,65,66,67,68,69]
Campylobacteriosis	Single strain: *B. pullicaecorum*, *L. salivarius* SMXD51 Compound: Multispecies lactic acid bacteria and prebiotic (synbiotic)	Compete for intestinal adhesion sites; produce antimicrobial metabolites; modulate cecal microbial structure	Reduce cecal *Campylobacter* load by up to 3 log_10_ CFU/g; significantly improve feed conversion efficiency	[46,70,71,72,73,74,75]
Avian pathogenic *E. coli* infection	Single strain: *E. faecium* NCIMB11181, *B. subtilis* Compound: Commercial multistrain probiotic mix	Repair intestinal tight junctions; suppress NF-κB inflammatory pathway; reduce bacterial translocation to visceral organs	Reduce mortality; upregulate *claudin-1* and *Muc2* expression; reverse elevated *E. coli* load in the liver	[76,77,78]

## Data Availability

No new data were created or analyzed in this study.
